# Spatiotemporal distribution of pregnancies among girls aged 14 years or younger in Bahia, Brazil, 2014–2023: an ecological study under the Brazilian legal framework of statutory rape

**DOI:** 10.1186/s12887-026-07250-2

**Published:** 2026-07-03

**Authors:** Rogério Ribeiro Ramos, Rita Carvalho-Sauer, Enny S. Paixao, Theolis Costa Barbosa Bessa

**Affiliations:** 1 Gonçalo Moniz Institute, Oswaldo Cruz Foundation, Salvador, Bahia Brazil; 2 East Regional Health Center, Health Department of the State of Bahia, Av. Esperança, 406, Santo Antônio de Jesus, Bahia Brazil; 3 London School of Hygiene & Tropical Medicine, London, UK

**Keywords:** Sexual violence against children, Statutory rape, Adolescent pregnancy, Spatiotemporal analysis, Health inequalities

## Abstract

**Background:**

Sexual violence against children is a severe violation of human rights and a persistent public health problem, with early pregnancy representing one of its most serious consequences. Although national studies in Brazil have identified geographic disparities in pregnancies resulting from statutory rape, intrastate heterogeneity remains insufficiently explored. This study aimed to describe the epidemiological profile of pregnancies among girls aged 14 years or younger interpreted under the Brazilian legal framework of statutory rape in the state of Bahia and to analyze their spatiotemporal distribution between 2014 and 2023.

**Methods:**

A spatiotemporal ecological study was conducted using secondary, aggregated data from the Live Birth Information System (SINASC) and the Mortality Information System (SIM), including live births and fetal deaths (≥ 22 weeks of gestation) among mothers aged ≤ 14 years residing in Bahia. Annual pregnancy rates per 1,000 female inhabitants aged 10–14 years were calculated by health region. Sociodemographic and clinical characteristics were described using absolute and relative frequencies. Spatiotemporal clusters with elevated risk were identified using Kulldorff’s scan statistic with a discrete Poisson model, retrospective analysis, 999 Monte Carlo replications, and a 5% significance level.

**Results:**

Between 2014 and 2023, 19,235 pregnancies were identified, including 18,914 live births and 321 fetal deaths. Most mothers self-identified as Brown, had low educational attainment, and were single. Preterm birth occurred in 17.5% and low birth weight in 14.0% of live-born infants. The statewide pregnancy rate declined from 3.5 to 2.5 per 1,000 girls aged 10 to 14 years (− 28.6%); however, marked regional disparities persisted. Four statistically significant spatiotemporal clusters were detected (*p* < 0.05), with relative risks ranging from 1.36 to 1.59, primarily concentrated in the South, Far South, North, and North-Central regions. Recurrent clusters across different periods indicated persistent territorial vulnerability.

**Conclusions:**

Despite an overall decline in rates, pregnancies among girls aged ≤ 14 years in Bahia, interpreted under the Brazilian legal framework of statutory rape, remain territorially concentrated, with persistent high-risk clusters. The persistence of high-risk clusters underscores the need for targeted, territorially tailored interventions integrating health care, surveillance, education, social assistance, and justice systems to prevent sexual violence and mitigate its reproductive consequences.

## Introduction

Sexual violence against children constitutes a severe violation of human rights and a highly complex public health issue [1, 2]. In Brazil, Article 217-A of the Penal Code defines statutory rape as any sexual act involving individuals under 14 years of age, regardless of the use of physical force, acknowledging that children and young adolescents lack the legal and developmental capacity to provide valid consent [3]. The biopsychosocial vulnerabilities inherent to prepubescent childhood amplify the harms caused by such violence, which extends across physical, psychological, social, and economic dimensions [2].

The magnitude of this problem is alarming. Worldwide, an estimated 11% of children experience sexual harassment, 9% suffer some form of sexual violence involving physical contact, and 6% are coerced into sexual intercourse at least once during their lifetime [4]. In Brazil, in 2024 alone, 87,545 rape cases were officially reported to the police, 77% of which involved children or adolescents [5]. These numbers likely underestimate the true burden, as sexual violence, particularly against minors, remains substantially underreported [5]. Among statutory rape victims, the majority were female (86.2%), identified as Black (55%) or White (43.1%), and most frequently aged 10–13 years (42.1%). Approximately 67.9% of incidents occurred in the victim’s home, and in 59.5% of cases the perpetrator was a family member [5].

The long-term consequences of sexual violence in childhood are profound, including depression, anxiety, post-traumatic stress disorder, eating disorders, suicidal ideation, substance abuse, chronic pain, sexual dysfunction, sexually transmitted infections, and early pregnancy [6]. Early pregnancy, in particular, disrupts school attendance, constrains social and economic opportunities, and contributes to the perpetuation of intergenerational cycles of vulnerability [7].

A recent national study examined live births to girls aged 10–14 years and six months in Brazil and identified spatial clusters of municipalities with elevated rates, particularly in the North, Midwest, and parts of the Northeast [8]. Although this study provided important evidence on the national distribution of the phenomenon, it was based exclusively on live births and was not designed to assess pregnancies ending in fetal deaths or to examine, in greater depth, the temporal persistence and recurrence of high-risk areas within individual states.Bahia, located in the Northeast region, exemplifies these territorial complexities. With 14.1 million inhabitants unevenly distributed across 417 municipalities and 28 health regions, the state presents important internal heterogeneity that may influence both the occurrence of pregnancies among girls aged 14 years or younger and access to health care, social protection, and justice services. In this context, a state-specific analysis may contribute to a more comprehensive understanding of the magnitude, distribution, and persistence of this phenomenon complementing national-level evidence and supporting more territorially responsive public policies.

Thus, the objective of this study was to describe the epidemiological profile of pregnancies among girls aged 14 years or younger in Bahia and to analyze the spatiotemporal distribution of these events from 2014 to 2023.

## Methods

### Study design and setting

This ecological spatiotemporal study used secondary, aggregated data on pregnancies resulting in live births or fetal deaths among girls aged 14 years or younger residing in Bahia, Brazil, from 2014 to 2023. The health region of residence was the geographic unit of analysis.

In Brazil, all births and deaths, including stillbirth, are subject to mandatory reporting [9]. However, fetal losses at < 22 weeks of gestation are not included in compulsory reporting, making their inclusion in the present study unfeasible.

### Data sources

Aggregated and anonymized data were obtained from routine health service records in information systems of the Brazilian Ministry of Health. From the Live Birth Information System (Sistema de Informações sobre Nascidos Vivos, SINASC), data was collected for mothers up to 14 years of age whose pregnancies resulted in live births. From the Mortality Information System (Sistema de Informações sobre Mortalidade, SIM), data were collected for mothers in the same age group whose pregnancies resulted in fetal death. Population estimates and projections for 2014–2023 from the Brazilian Institute of Geography and Statistics (IBGE) were also retrieved for calculating rates [10, 11]. The Brazilian Federal Government provides these and other datasets on the Open Data Portal of the Department of Informatics of the Unified Health System < https://dadosabertos.saude.gov.br/%3E>.

### Study population and eligibility criteria

The study included maternal data from all live birth and fetal death certificates recorded between 2014 and 2023 in which the mother was aged 14 years or younger at the end of pregnancy. Pregnancies ending in fetal loss before 22 weeks of gestation were not included, as these events are not subject to compulsory notification in Brazilian national health information systems.

Pregnancies in this age group were interpreted according to the Brazilian legal framework on statutory rape. Under Article 217-A of the Brazilian Penal Code [3], sexual intercourse or any libidinous act with a person under 14 years of age constitutes statutory rape, regardless of consent or the use of physical force. Girls aged 14 years at the end of pregnancy were retained because, given the duration of pregnancy, conception may have occurred before the age of 14. However, exact age at conception was not available in the aggregated data.

SINASC and SIM do not record the circumstances of conception, the existence of coercion, police reporting, judicial confirmation, or the relationship between the child and the perpetrator. Therefore, this study does not ascertain individual-level causality or documented sexual violence in each case. Rather, it analyzes pregnancies in this legally vulnerable age group as events that fall within, or are closely related to, the Brazilian legal definition of statutory rape, acknowledging this premise as a methodological limitation.

### Definitions

A live birth was defined as any product of conception that, after complete expulsion or extraction from the mother’s body, regardless of gestational age, breathed or showed any other sign of life, such as heartbeat, umbilical cord pulsation, or voluntary muscle movement [12].

A fetal death was defined as a product of conception with no signs of life before complete expulsion or extraction, with birth weight ≥ 500 g, gestational age ≥ 22 weeks, or crown–heel length ≥ 25 centimeters [12].

### Variables and descriptive analysis

To describe the epidemiological profile of the study participants, absolute and relative frequencies of sociodemographic and clinical variable categories were calculated and presented in tabular format.

### Rate calculation

To characterize the spatiotemporal distribution of cases, the annual rate of pregnancies in mothers aged 14 years or younger per 1,000 female inhabitants aged 10–14 years was calculated according to the health region of residence.

First, the number of live births and fetal deaths of mothers aged 14 years or younger was summed by place of residence and year of occurrence. The resulting value was then divided by the population of girls aged 10–14 years residing in the same location and period and multiplied by 1,000. The historical series of these rates from 2014 to 2023 was presented in tabular form. 

The Ministry of Health (2009) defines “women of reproductive age” as those aged 10–49 years [13]. For this reason, the population of female residents aged 10–14 years was used as the denominator for calculating pregnancy rates among mothers up to 14 years.

Although one case of pregnancy in a child under 10 years of age was recorded during the study period, this extremely small number over a 10-year interval did not justify the inclusion of girls under 10 in the population denominator. Doing so would have diluted rates and potentially masked the true magnitude of the problem.

### Spatiotemporal analysis

Kulldorff’s spatiotemporal scan statistic [14] was employed to detect high-incidence clusters of pregnancies in mothers up to 14 years of age across space and time in Bahia, from 2014 to 2023.

This method involves moving virtual cylinders across space and time to identify spatiotemporal clusters assuming a Poisson distribution, with estimation of the relative risk (RR) for each cluster [14, 15]. The discrete Poisson probability model is considered appropriate for rare events and data aggregated by geographic units.

The observed number of cases inside each cylinder is compared with the expected number, given the underlying population. Neighboring areas whose centroids fall within the cylinder’s base are included in the cluster. Under the null hypothesis, the risk inside the cylinder is equal to the risk outside [14, 15].

A retrospective scan was conducted, with circular spatiotemporal windows exploring all possible combinations of location and time, identifying clusters with higher-than-expected rates at a 5% significance level [14, 15]. 

Data were aggregated by the 28 health regions of the state of Bahia, using geographic coordinates defined by the latitude and longitude of each region’s centroid, and the temporal unit was the year. 

Data inputs included: (i) number of pregnancies in mothers aged 14 years or younger by health region and year; (ii) population of female residents aged 10–14 years by health region and year; and (iii) geographic coordinates of health regions. 

The study period spanned January 1, 2014 to December 31, 2023. The maximum spatial cluster size was set at 50% of the population at risk, and the temporal window ranged from a minimum of 1 to a maximum of 5 years (50% of the study duration). No nonparametric temporal trend was considered. A total of 999 Monte Carlo replications were performed to assess the statistical significance of detected clusters. Only non-overlapping clusters with significantly elevated relative risk (*p* < 0.05) were reported. Clusters were described according to the log-likelihood ratio (LLR), with the cluster showing the highest LLR considered the least likely to have occurred by chance.

### Software

SaTScan™ (v. 10.2.1) [16] and QGIS (v. 3.34.6) [17] were used, respectively, to perform spatiotemporal analyses and produce maps.

## Results

Between 2014 and 2023, 19,235 pregnancies among girls aged 14 or younger were recorded in Bahia, 18,914 resulted in live births, and 321 resulted in fetal deaths. Most of these young mothers self-identified as Brown (75.28%); however, information on race/skin color was not available for fetal death records. Regarding education, the majority (68.41%) had completed up to elementary school, and most records with available information indicated single marital status (75.47%). The number of prenatal consultations ranged from 4 to 6 (35.15%) and 7 or more (46.60%) for pregnancies resulting in live births, but this information was unavailable for pregnancies ending in fetal death. Gestational duration was ≤ 36 weeks for 18.45%, and 28.65% of deliveries were cesarean sections. Among live births, 13.95% presented low birth weight, and 51.46% of newborns were male (Table 1).

**Table 1:** Sociodemographic and clinical characteristics of mothers aged ≤ 14 years, according to pregnancy outcome. Bahia, 2014–2023

	Gestational outcome				Total	
	Live-born (*N* = 18.914)		Fetal death (*N* = 321)		(*N* = 19.235)	
Sociodemographic and clinical characteristics	*N*	%	*N*	%	*N*	%
*Mother’s Race / Color ¹*						
White	927	4.90	-	-	927	4.82
Black	2,022	10.69	-	-	2,022	10.51
Brown	14,481	76.56	-	-	14,481	75.28
Asian	72	0.38	-	-	72	0.37
Indigenous	184	0.97	-	-	184	0.96
Missing	1,228	6.49	321	100	1,549	8.05
*Education level*						
No schooling	46	0.24	7	2.18	53	0.28
Incomplete elementary school	12,751	67.42	229	71.34	12,980	67.48
Complete elementary school	5,133	27.14	43	13.40	5,179	26.91
Missing	984	5.20	42	13.08	1,026	5.33
*Mother’s marital status ¹*						
Married	154	0.81	-	-	154	0.80
Legally separated	9	0.05	-	-	9	0.05
Single	14,516	76.75	-	-	14,516	75.47
Widowed	2	0.01	-	-	2	0.01
Other	3,720	19.67	-	-	3,720	19.34
Missing	513	2,71	321	100	834	4.34
*Number of prenatal visits ¹*						
None	765	4.04	-	-	765	3.98
1 to 3	2,214	11.71	-	-	2,214	11.51
4 to 6	6,762	35.75	-	-	6,762	35.15
7 or more	8,964	47.39	-	-	8,964	46.60
Missing	209	1.11	321	100	530	2.76
*Duration of pregnancy*						
≤ 36 weeks	3,306	17.48	243	75.7	3,549	18.45
≥ 37 weeks	14,679	77.61	47	14.6	14,726	76.56
Missing	929	4.91	31	9.7	960	4.99
*Mode of delivery*						
Vaginal	13,407	70.88	281	87.5	13,688	71.16
Cesarean	5,475	28.95	35	10.9	5,510	28.65
Missing	32	0,17	5	1.6	37	0.19
*Baby’s sex*						
Male	9,741	51,50	157	48.9	9,898	51.46
Female	9,168	48,47	148	46.1	9,316	48.43
Missing	5	0,03	16	5.0	21	0.11
*Baby’s birth weight*						
< 2500 g	2,639	13,95	257	80.1	2,896	15.06
≥ 2500 g	16,270	86,02	49	15.3	16,319	84.84
Missing	5	0,03	15	4.7	20	0.10

^1^variable unavailable in fetal death records

Source: Live Birth Information System (SINASC/DATASUS); Mortality Information System (SIM/DATASUS). Accessed on September 14, 2024

The rate of pregnancies among mothers aged 14 years or younger in Bahia per 1,000 female inhabitants aged 10 to 14, between 2014 and 2023, declined from 3.5 to 2.5 over the study period, however revealed marked regional variations. The regions of Ilhéus, Itabuna, Valença, and Juazeiro showed persistently high rates over the years, frequently exceeding 4.5 per 1,000 girls. In contrast, the regions of Salvador and Seabra maintained the lowest rates, with an apparent downward trend. In terms of percentage variation, substantial negative fluctuations were identified in regions such as Camaçari (-51.28%), Salvador (-62.07%), and Seabra (-64.10%). These declines contrast with the absence of change observed in Senhor do Bonfim (0.0%) and Serrinha (0.0%), as well as with the increases recorded in Juazeiro (11.76%) and Ibotirama (56.0%) in the rates of births among girls under the age of 14, per 1,000 girls aged 10 to 14, when comparing the years 2014 and 2023. Table 2 illustrates the temporal and spatial distribution of these rates, respectively. A decreasing trend is evident over time, although some areas continue to sustain high levels (Table 2).

**Table 2:**
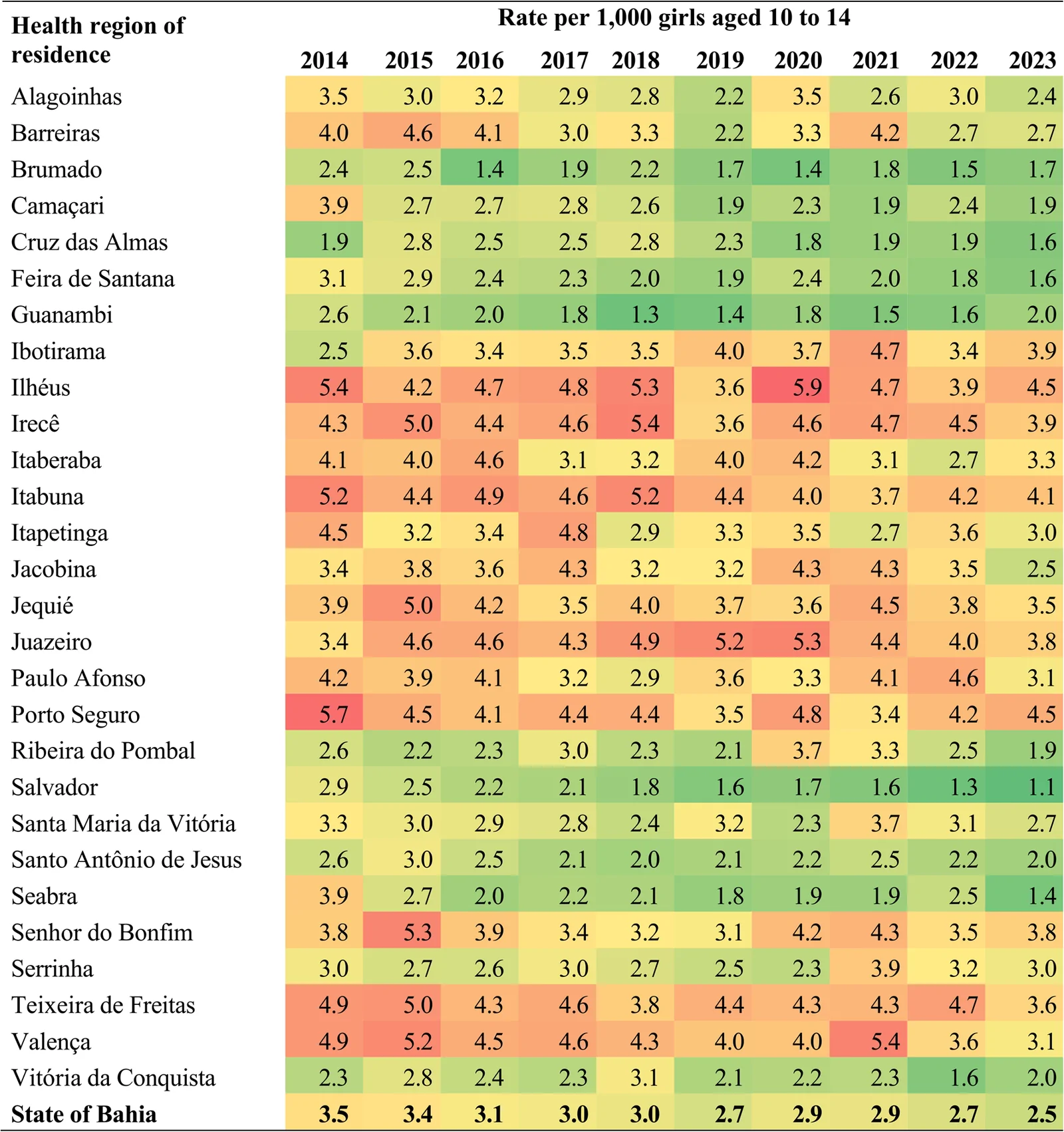
Pregnancy rate among mothers aged ≤ 14 years per 1,000 female inhabitants aged 10–14, according to health region of residence. Bahia, 2014–2023

Source: Live Birth Information System (SINASC/DATASUS); Mortality Information System (SIM/DATASUS). Brazilian Institute of Geography and Statistics (IBGE). Accessed on September 14, 2024. Health regions are presented in alphabetical order to facilitate identification across the ten-year time series

Values in bold correspond to the rates for the state of Bahia as a whole

**Fig. 1:**
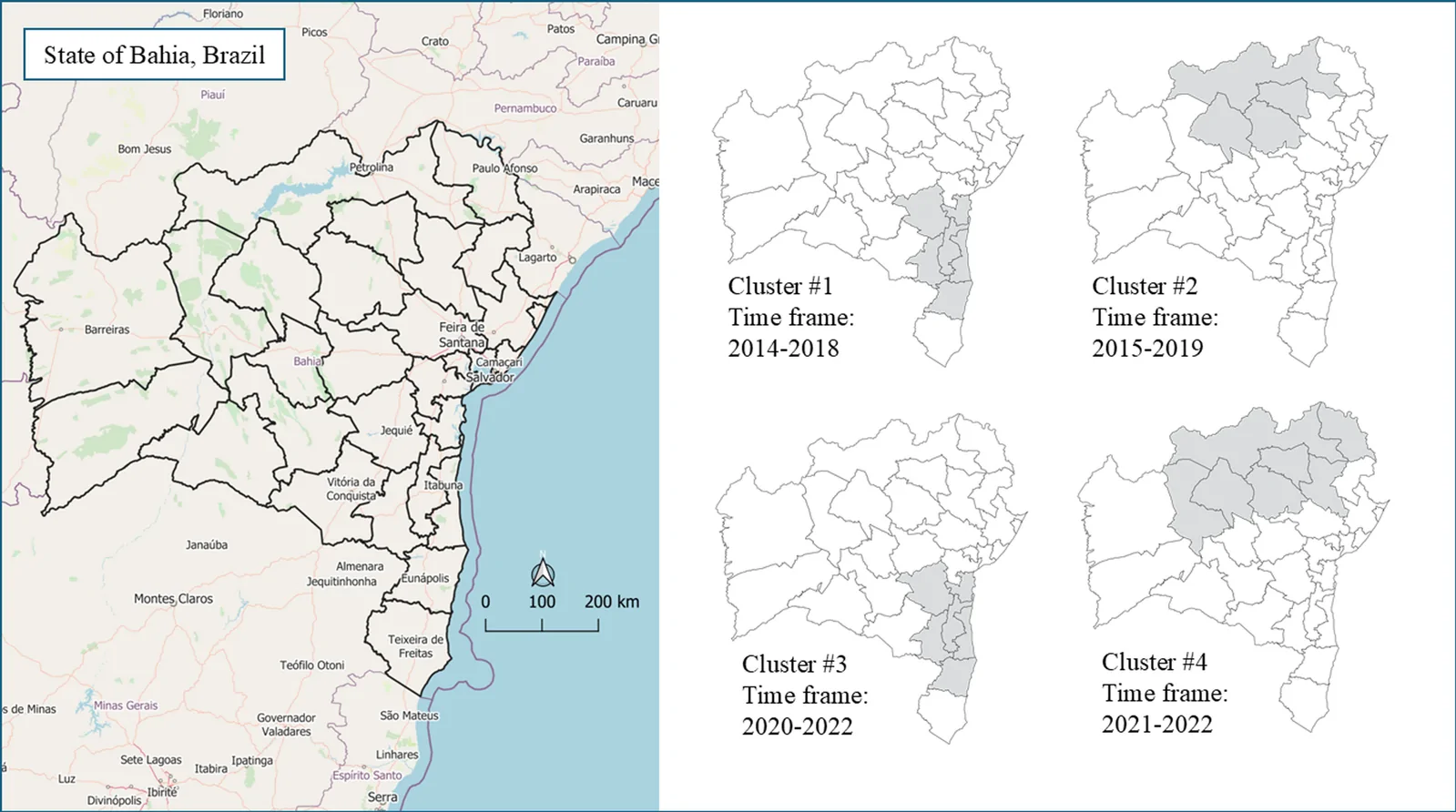
Statistically significant spatiotemporal clusters of health regions with high incidence of pregnancies among mothers aged ≤ 14 years per 1,000 female inhabitants aged 10–14, identified using Kulldorff’s scan statistic. Bahia, 2014–2023. Source: Prepared by the authors

The spatiotemporal analysis identified four statistically significant clusters (*p* < 0.05) with elevated risk of pregnancies among girls aged 14 years or younger in Bahia between 2014 and 2023 (Fig. 1).

The most likely cluster (cluster #1) was concentrated in the health regions of Ilhéus, Itabuna, Itapetinga, Jequié, Porto Seguro, and Valença. With a centroid located at 15.13°S and 39.21°W and a radius of 191.16 km, this cluster was active from 2014 to 2018. A total of 2,566 cases were recorded, compared to 1,699 expected, resulting in a relative risk (RR) of 1.59 (*p* < 0.0001). Secondary cluster #2 encompassed the regions of Irecê, Jacobina, Senhor do Bonfim, and Juazeiro, with a centroid at 9.73°S and 41.39°W and a radius of 192.84 km. Identified between 2015 and 2019, this cluster showed an RR of 1.48 (*p* < 0.0001). Cluster #3 revealed the persistence of the spatial pattern observed in cluster #1, covering the same health regions (Ilhéus, Itabuna, Itapetinga, Jequié, Porto Seguro, and Valença), but in a later period, from 2020 to 2022. There were 1,002 observed cases, compared to 745 expected, resulting in an RR of 1.36 (*p* < 0.0001). Cluster #4 comprised an area that included the regions of Juazeiro, Senhor do Bonfim, Irecê, Jacobina, Serrinha, Ibotirama, and Paulo Afonso. With a radius of 295.92 km, this cluster was active between 2021 and 2022, totaling 829 observed cases versus 614 expected, with an RR of 1.37 (*p* < 0.0001) (Table 3).

**Table 3:** Characteristics of the spatiotemporal clusters of health regions with high incidence of pregnancies among mothers aged ≤ 14 years per 1,000 female inhabitants aged 10–14, identified using Kulldorff’s scan statistic. Bahia, 2014–2023

Variables	Cluster number			
	#1	#2	#3	#4
Coordinates	15.132201 S, 39.213545 W	9.727567 S, 41.387786 W	15.132201 S, 39.213545 W	9.727567 S, 41.387786 W
Health Regions included	Ilhéus, Itabuna, Itapetinga, Jequié, Porto Seguro, and Valença	Irecê, Jacobina, Senhor do Bonfim, and Juazeiro	Ilhéus, Itabuna, Itapetinga, Jequié, Porto Seguro, and Valença	Juazeiro, Senhor do Bonfim, Irecê, Jacobina, Serrinha, Ibotirama, and Paulo Afonso
Time frame	01/01/2014 to 12/31/2018	01/01/2015 to 12/31/2019	01/01/2020 to 12/31/2022	01/01/2021 to 12/31/2022
Radius (km)	191.16	192.84	191.16	295.92
Span (km)	297.82	227.38	297.82	545.11
Population	99,911	75,586	99,911	125,904
Number of cases	2566	1796	1002	829
Expected cases	1699.23	1254.93	744.63	613.71
Observed / expected	1.51	1.43	1.35	1.35
Relative risk	1.59	1.48	1.36	1.37
Log likelihood ratio	212.99	111.11	41.91	35.25
*p*-value	**< 0.0000001**	**< 0.0000001**	**< 0.0000001**	**< 0.0000001**

## Discussion

This study showed that, although the statewide rate of pregnancies among girls aged ≤ 14 years, interpreted under the Brazilian legal framework of statutory rape, declined by 28.57% in Bahia between 2014 and 2023, from 3.5 to 2.5 per 1,000 girls aged 10–14 years, this reduction was not homogeneous across the state. Some health regions experienced more pronounced declines, whereas others showed stability or even increases over the 10-year period. The identification of statistically significant spatiotemporal clusters further indicates that certain territories sustained consistently elevated rates over time, reinforcing the role of territorial inequalities in shaping the distribution of this public health problem.

The sociodemographic profile of the cases should be interpreted within this broader context and with appropriate caution. Most girls had incomplete or complete elementary education and were single, findings that are largely expected given the young age of the study population and therefore should not be interpreted, in isolation, as differentiating markers of vulnerability. However, pregnancy at such an early age may interrupt educational trajectories, restrict future social and economic opportunities, and contribute to the perpetuation of intergenerational cycles of vulnerability [7]. Similarly, the predominance of girls self-identified as Brown or Black must be understood in light of the demographic composition of Bahia and the racial and social inequalities that historically shape access to education, protection networks, health care, and reproductive rights in Brazil [18, 19].

Although these characteristics are relevant for contextualizing the phenomenon and are consistent with national evidence on the intersection of gender, race/skin color, and socioeconomic inequality [5, 8], the present ecological study did not estimate race-, education-, or marital-status-specific risks. Thus, these variables should be understood primarily as part of the descriptive epidemiological profile of pregnancies in this legally vulnerable age group.

Beyond sociodemographic characteristics, the high proportion of pregnancies with fewer than seven prenatal visits, as well as the frequencies of preterm birth and low birth weight, point to important health care and reproductive vulnerabilities among girls experiencing pregnancy at very young ages. In many situations, the child and her family may initially struggle to recognize or disclose the pregnancy, often amid fear, shame, or family and social constraints, which can delay the initiation of prenatal care [20, 21]. These findings reinforce the need for timely identification, comprehensive care, and intersectoral protection responses [22, 23].

The identification of recurrent clusters, that is, groupings comprising the same regions across different time periods, suggests the presence of underlying structural factors beyond temporal fluctuations. This pattern was observed for clusters #1 and #3, which consisted of the same health regions and emerged as high-risk clusters between 2014 and 2018, and again from 2020 to 2022; as well as for cluster #2, which emerged as a high-risk grouping between 2015 and 2019 and subsequently reemerged, encompassing the same regions along with additional health regions (cluster #4) between 2021 and 2022. This pattern suggests that persistent local contextual factors may be contributing to the sustained burden of this public health problem [24–26]. This highlights the urgent need for targeted interventions in these specific areas, which include territories characterized by longstanding socioeconomic inequalities, extensive rural territories, limited access to specialized services, and fragile child and adolescent protection networks, conditions that may facilitate both the occurrence of sexual violence and barriers to timely care for the resulting pregnancy [27–29].

The overall decline in rates across the historical series may reflect gradual advances in policies to address sexual violence, expanded access to education, and increased availability of contraceptive methods. However, the persistence and, in some instances, intensification of clusters in more recent periods, including 2020–2022, may be partly related to indirect impacts of the COVID-19 pandemic, including school closures, prolonged household confinement, reduced access to health and social protection services, and fewer opportunities for disclosure and reporting; factors described in the literature as amplifying risks of sexual violence against children and adolescents [30–32].

Our findings are consistent with national analyses reporting higher rates of pregnancy among girls younger than 14 years in Brazil’s North and Northeast regions [8], while extending this evidence by demonstrating pronounced intrastate heterogeneity. By using intermediate territorial units (health regions), the present analysis captured patterns that may not be detectable in national-level studies, thereby offering greater applicability for state-level planning of health and social protection actions.

Overall, our results reinforce the need for territorialized strategies that integrate epidemiological surveillance, the health care network, the education system, social assistance, and the justice sector. Regions identified as priorities should receive coordinated interventions, including strengthening primary health care, training professionals for early identification of sexual violence, ensuring timely access to emergency contraception and legal abortion, and implementing intersectoral policies to support school retention and protect girls in situations of vulnerability. In addition, spatiotemporal analysis emerges as a strategic tool to inform planning, enabling more efficient allocation of resources and efforts.

Some limitations should be considered when interpreting our results. Although Brazilian law [3] defines sexual acts with individuals under 14 years of age as statutory rape, the information systems used in this study do not record the circumstances of conception, coercion, police investigation, judicial confirmation, or the relationship between the child and the perpetrator. In addition, for girls aged 14 years at the end of pregnancy, the classification assumes that conception generally occurred before the age of 14, which could not be verified using the aggregated data available. Therefore, the findings should be interpreted as describing pregnancies in a legally vulnerable age group, rather than as individual confirmation of documented sexual violence in each case.

The use of secondary data is also subject to underreporting and inconsistencies in record completion, particularly for sensitive variables such as race/skin color. Moreover, pregnancies ending in abortion or fetal loss before 22 weeks of gestation were not included, likely leading to underestimation of the true magnitude of pregnancies in this age group.

Although the spatiotemporal scan statistic is suitable for detecting high-risk clusters, this study did not estimate adjusted associations with contextual determinants, such as socioeconomic, demographic, or service-related indicators; future analyses using Bayesian or other hierarchical spatiotemporal models may help explain the mechanisms underlying the observed territorial patterns.

Finally, because this is an ecological study based on aggregated data at the health-region level, the findings are subject to ecological fallacy and should not be interpreted as individual-level risk estimates or causal associations, but rather as territorial patterns of concentration useful for guiding surveillance, planning, and intersectoral public health responses.

Despite these limitations, this study has important strengths. It relied on robust and reliable databases with universal, mandatory reporting across public and private health services and near-complete coverage [10]. It also addressed the problem more comprehensively by including not only pregnancies resulting in live births but also those ending in fetal deaths at ≥ 22 weeks of gestation.

Collectively, these findings provide evidence that pregnancy among girls aged ≤ 14 years, interpreted according to the Brazilian legal framework on statutory rape, remains a territorially concentrated and unequal phenomenon in Bahia. The implementation of structured, sustained responses tailored to local specificities is urgently needed to prevent the perpetuation of cycles of violence, exclusion, and intergenerational inequalities.

## Conclusions

Despite a substantial decline in the statewide rate of pregnancies among girls aged ≤ 14 years, interpreted according to the Brazilian legal framework on statutory rape, in Bahia between 2014 and 2023, this study demonstrates that the burden of this phenomenon remains unequally distributed, with persistent high-risk clusters concentrated in specific territories. The identification of recurrent spatiotemporal clusters highlights the need for sustained, territorially targeted, and intersectoral responses. Strengthening integrated actions across health, education, social protection, and justice systems is essential to prevent sexual violence, ensure timely and comprehensive care for affected girls, and mitigate adverse reproductive outcomes. Spatiotemporal analytical approaches can play a role in guiding these efforts, supporting more equitable allocation of resources and interventions.

## Data Availability

All data used in this study are publicly available and fully anonymized. The datasets analyzed can be accessed from open sources without restrictions. Links to the original repositories or platforms are: 1 Live births: TabNet Win32 3.0: NASCIDOS VIVOS - BAHIA. 2 Fetal deaths: TabNet Win32 3.0: Óbitos Fetais - Bahia. 3 Population: TabNet Win32 3.0: POPULAÇÃO RESIDENTE ESTIMADA, ESTRATIFICADA POR SEXO E FAIXA ETÁRIA - BAHIA.

## Abbreviations

IBGE: Brazilian Institute of Geography and Statistics
LLR: Log–likelihood ratio
RR: Relative risk
SIM: Mortality Information System
SINASC: Live Birth Information System
